# Krill Oil Inhibits Cholesterol Synthesis and Stimulated Cholesterol Excretion in Hypercholesterolemic Rats

**DOI:** 10.3390/md20100609

**Published:** 2022-09-27

**Authors:** Ok-Kyung Kim, Jeong Moon Yun, Dakyung Kim, Soo-Jeung Park, Chungil Lee, Eun Byeol Go, Jae Sil Kim, Sang Yong Park, Jeongmin Lee

**Affiliations:** 1 Division of Food and Nutrition, Human Ecology Research Institute, Chonnam National University, Gwangju 61186, Korea; 2 Department of Medical Nutrition, Kyung Hee University, Yongin 17104, Korea; 3 Beirne B. Carter Center for Immunology Research, University of Virginia, Charlottesville, VA 22908, USA; 4 SD Biotechnologies Co., Ltd., Seoul 07793, Korea; 5 Research Institute of Clinical Nutrition, Kyung Hee University, Seoul 02447, Korea

**Keywords:** hypercholesterolemia, krill oil, cholesterol

## Abstract

The present study aimed to investigate the antihypercholesterolemic effects of krill oil supplementation in high-cholesterol diet-induced hypercholesterolemic rats, and the mechanisms underlying these effects. Rats were divided into five groups: normal control, control (high-cholesterol diet), krill oil 100 mg/kg b.w. (high-cholesterol diet with Krill oil 100 mg/kg b.w.), and krill oil 200 mg/kg b.w. (high-cholesterol diet with Krill oil 200 mg/kg b.w.). After 12 weeks, the rats were sacrificed to observe the effects of krill oil on cholesterol synthesis and excretion. We found that krill oil supplementation suppressed total triglycerides, total cholesterol, and LDL-cholesterol levels, as well as HMG-CoA reductase activity. It stimulated AMPK phosphorylation, LDL receptor and ACAT2 expression in the liver, and the fecal output of cholesterol. Furthermore, it decreased the levels of P-selectin, sVCAM-1, and NO, as well as aortic wall thickness, demonstrating its role in the prevention of atherosclerosis. Thus, we suggest that krill oil supplementation can reduce LDL-cholesterol levels in the blood during hypercholesterolemia by stimulating the uptake of LDL-cholesterol into tissue and cholesterol excretion, as well as inhibition of cholesterol synthesis.

## 1. Introduction

Cholesterol is essential for maintaining the integrity and fluidity of cell membranes and metabolic processes, including the production of steroid hormones, bile acids, and vitamin D [1]. However, hypercholesterolemia, which is defined as elevated levels of low-density lipoprotein (LDL) cholesterol, is an important risk factor for atherosclerotic cardiovascular disease [1,2]. Increased LDL-cholesterol levels lead to the formation of plaque by LDL-cholesterol oxidation-induced macrophage foam cells, thereby triggering the development of atherosclerosis. Cholesterol homeostasis is, therefore, important in maintaining appropriate cellular function and preventing atherosclerotic cardiovascular disease [3,4].

Cholesterol homeostasis in cell maintenance is tightly regulated by both intracellular and extracellular cholesterol; the process involves the uptake of cholesterol into cells, biosynthesis, esterification for storage, and excretion into bile acids. When excess cholesterol is present in cells, uptake of LDL-cholesterol is decreased by inhibiting the expression of the LDL receptor; the excess cholesterol is esterified by cholesterol acyltransferase (ACAT), resulting in elevated LDL-cholesterol in blood [5,6]. When low cholesterol is present in cells, it can be biosynthesized by 3-hydroxy-3-methyl-glutaryl-coenzyme A (HMG-CoA) reductase, which is the rate-limiting enzyme in cholesterol synthesis [7]. The synthesis and excretion of bile acids in the liver is the main pathway involved in excretion of excess cholesterol; however, most bile acids are reabsorbed in the ileum [8]. Therefore, blood cholesterol can be reduced via inhibition of HMG-CoA reductase activity and bile acid reabsorption [5,6,7,8,9].

The causal relationship between hypercholesterolemia and atherosclerosis is well known; thus, the evaluation of dyslipidemia, including hypercholesterolemia, plays a pivotal role in the assessment of cardiovascular disease. Therefore, patients with hypercholesterolemia should be prescribed medication to decrease LDL-cholesterol and reduce the risk of cardiovascular disease [10]. Conventional prescribed pharmacological drugs include statins (HMG-CoA reductase inhibitors), ezetimibe (blocking cholesterol absorption), and bile acid sequestrants (bile acid-binding agents to get rid of cholesterol). Nevertheless, these drugs produce side effects, including liver damage, muscle inflammation, type 2 diabetes, diarrhea, and abdominal pain. Therefore, alternative therapies were used in an attempt to treat hypercholesterolemia, including natural cholesterol-lowering agents, such as niacin, soluble fiber, and plant sterols, which have a pharmacological effect similar to that of the drugs [11,12,13]. Recently, several studies have demonstrated that krill oil [extracted from Antarctic krill (*Euphausia superba*)] supplementation can act as an antihyperlipidemic agent. However, the exact mechanism of its effect was not identified. Krill oil contains abundant omega-3 (n−3) long-chain polyunsaturated fatty acids (LC-PUFA) which are excellent sources of both eicosapentaenoic acid (20:5n−3, EPA) and docosahexaenoic acid (C22:6n−3, DHA) [14,15,16]. Numerous studies have investigated the roles of n-3 LC-PUFA in many metabolic and physiological processes and effects in the prevention of cardiovascular decrease [17,18]. In the present study, we investigated the antihypercholesterolemic effects of krill oil (obtained by enzymic hydrolysis from krill) supplementation by observing its effect on cholesterol synthesis and excretion in high-cholesterol diet-induced rats.

## 2. Results

### 2.1. Krill Oil Protected against Liver Damage and Improved Lipid Profile in Hypercholesterolemic Rats

We found that the high-cholesterol-diet control group had increased liver weight and serum levels of ALT and AST compared with those in the normal diet control group (NC), indicating the development of liver damage or hepatotoxicity. However, the high-cholesterol diet with krill oil 200 mg/kg b.w. group (KO–H) revealed a significant decrease in liver weight and serum levels of ALT and AST compared with those in the high-cholesterol diet group (*p* < 0.05) (Table 1).

**Figure 1:**
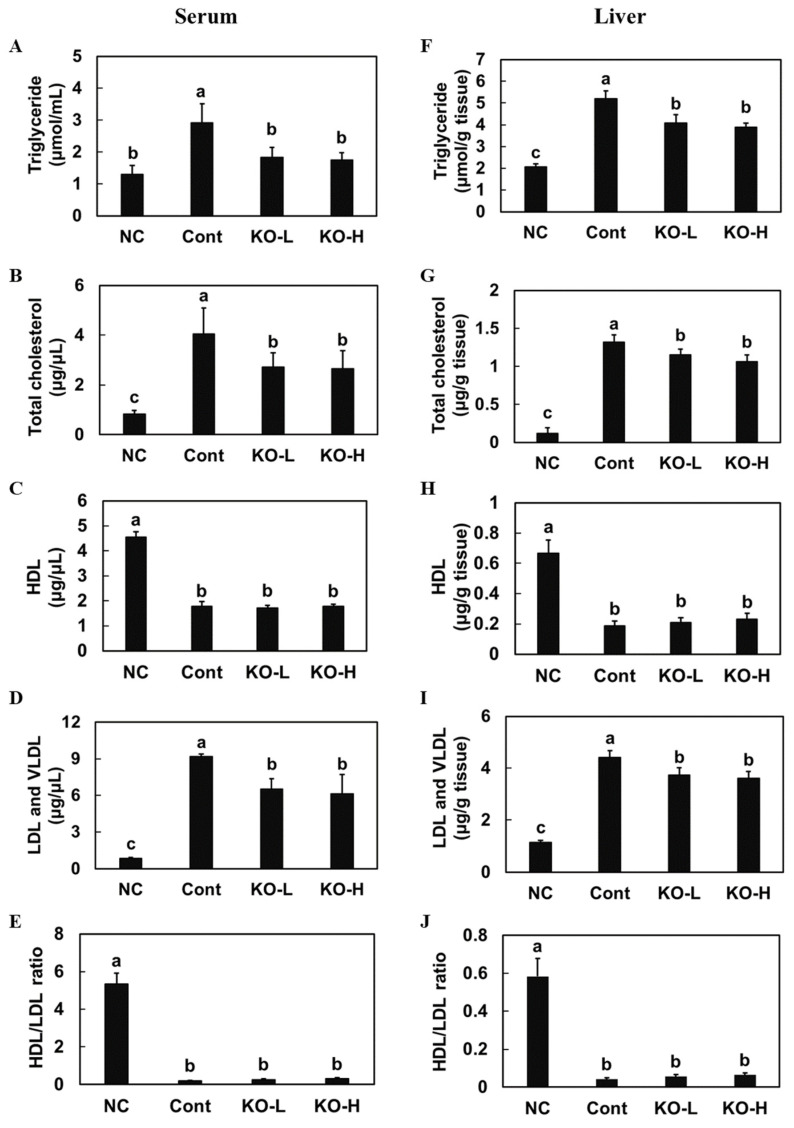
Change in lipid profiles of serum and liver from hypercholesterolemic rats fed a diet supplemented with krill oil. Levels of triglyceride (**A**), total cholesterol (**B**), HDL (**C**), LDL and VLDL (**D**), and HDL/LDL ratio (**E**) in the serum and triglyceride (**F**), total cholesterol (**G**), HDL (**H**), LDL and VLDL (**I**), and HDL/LDL ratio (**J**) in the liver. NC, normal AIN93G diet control group; Cont, high-cholesterol diet control group; KO–L, high-cholesterol diet with krill oil 100 mg/kg b.w.; KO–H, high-cholesterol diet with krill oil 200 mg/kg b.w. Values are presented as means ± SD. Different letters indicate a significant difference at *p* < 0.05, as determined by Duncan’s multiple range test.

The high-cholesterol diet control group had increased total triglycerides, total cholesterol, and LDL-cholesterol levels, but decreased HDL-cholesterol levels in both serum and liver compared with those in the normal diet control group; however, the high-cholesterol diet with krill oil 100 mg/kg b.w group (KO–L) and KO–H groups had decreased total triglycerides, total cholesterol, and LDL-cholesterol levels compared with those in the high-cholesterol diet group (*p* < 0.05) (Figure 1). These results suggest that krill oil supplementation protected against liver damage and improved lipid profile in rats receiving a high-cholesterol diet.

### 2.2. Krill Oil Inhibited Cholesterol Synthesis and Stimulated Cholesterol Uptake in the Liver of Hypercholesterolemic Rats

**Figure 2:**
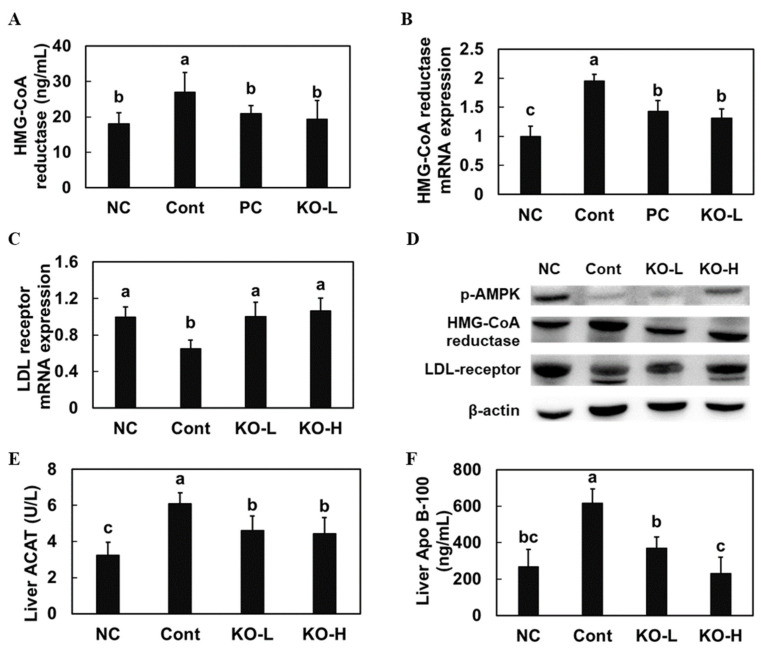
HMG-CoA reductase activity (**A**), HMG-CoA reductase mRNA expression (**B**), LDL mRNA expression (**C**), LDL protein expression (**D**), ACAT2 level (**E**), and Apo B-100 level (**F**), in the liver from hypercholesterolemic rats fed a diet supplemented with krill oil. NC, normal AIN93G diet control group; Cont, high-cholesterol-diet control group; KO–L, high-cholesterol diet with krill oil 100 mg/kg b.w.; KO–H, high-cholesterol diet with krill oil 200 mg/kg b.w. Values are presented as means ± SD. Different letters indicate a significant difference at *p* < 0.05, as determined by Duncan’s multiple range test.

To confirm the effects of krill oil on cholesterol synthesis, we measured HMG-CoA reductase activity (Figure 2A), mRNA expression (Figure 2B), and protein expression (Figure 2D and Figure S1) in the liver of hypercholesterolemic rats. The high-cholesterol-diet control group showed increased HMG-CoA reductase activity, mRNA expression, and protein expression compared with those aspects in the normal diet control group. Moreover, the high-cholesterol-diet control group presented decreased AMPK phosphorylation in the liver (Figure 2D and Figure S1); however, krill oil supplementation groups showed decreased HMG-CoA reductase activity, mRNA expression, and protein expression, and increased AMPK phosphorylation compared with those aspects in the high-cholesterol-diet control group (*p* < 0.05).

A high-cholesterol diet induced a decrease in the mRNA and protein expression of the LDL receptor in the liver of hypercholesterolemic rats compared with that in the normal diet control group; however, krill oil supplementation groups showed an increase in the mRNA and protein expression of the LDL receptor compared with that in the high-cholesterol-diet control group (Figure 2C). Furthermore, hepatic cholesterol ACAT2 and ApoB protein levels were significantly increased in the high-cholesterol-diet control group compared with that in the normal diet control group; however, they were significantly decreased in the krill oil supplementation groups compared with those in the high-cholesterol-diet control group (*p* < 0.05) (Figure 2E,F).

### 2.3. Krill Oil Stimulated the Excretion of Cholesterol and Bile Acid in Hypercholesterolemic Rats

**Figure 3:**
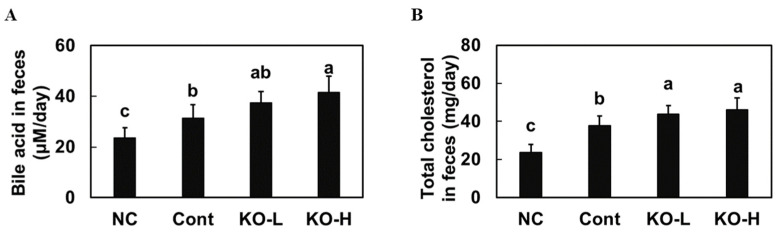
Level of bile acid (**A**) and total cholesterol (**B**) in feces from hypercholesterolemic rats fed a diet supplemented with krill oil. NC, normal AIN93G diet control group; Cont, high-cholesterol-diet control group; KO–L, high-cholesterol diet with krill oil 100 mg/kg b.w.; KO–H, high-cholesterol diet with krill oil 200 mg/kg b.w. Values are presented as means ± SD. Different letters indicate a significant difference at *p* < 0.05, as determined by Duncan’s multiple range test.

We measured the total cholesterol and bile acid in feces of hypercholesterolemic rats to confirm whether krill oil supplementation affects cholesterol excretion. A high-cholesterol diet caused a significant increase in the total cholesterol and bile acid levels in the feces of hypercholesterolemic rats compared with those in the normal diet control group. Krill oil supplementation significantly increased the total cholesterol and bile acid levels in the feces of hypercholesterolemic rats compared with those in the high-cholesterol-diet control group (*p* < 0.05) (Figure 3). Thus, we suggest that krill oil supplementation stimulated the excretion of cholesterol and bile acid in hypercholesterolemic rats.

### 2.4. Krill Oil Decreased Atherosclerotic Wall Thickness

**Figure 4:**
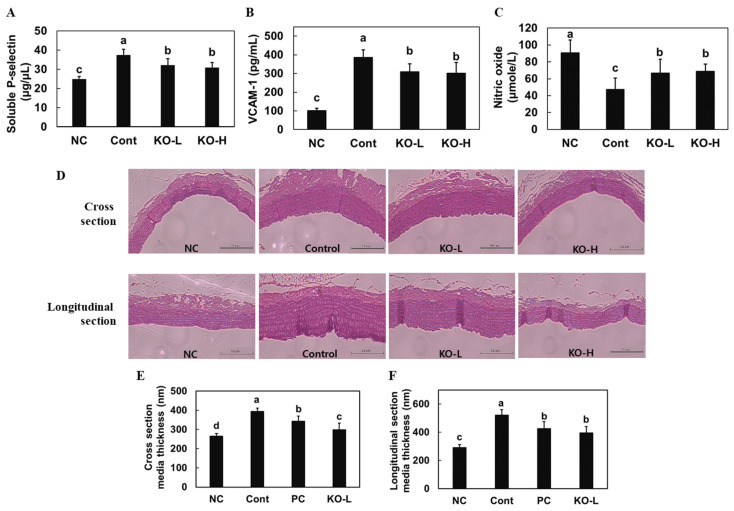
Levels of P-selectin (**A**), sVCAM-1(**B**), and nitric oxide (**C**) in serum and aortic wall thickness (**D**–**F**) in hypercholesterolemic rats fed a diet supplemented with krill oil. NC, normal AIN93G diet control group; Cont, high-cholesterol diet control group; KO–L, high-cholesterol diet with krill oil 100 mg/kg b.w; KO–H, high-cholesterol diet with krill oil 200 mg/kg b.w. Values are presented as means ± SD. Different letters indicate a significant difference at *p* < 0.05, as determined by Duncan’s multiple range test.

The levels of P-selectin, sVCAM-1, and NO in serum were significantly increased in the high-cholesterol-diet group compared with those in the normal diet group (Figure 4A–C). Moreover, the high-cholesterol diet induced a significant increase in the aortic wall thickness compared with that by the normal diet; however, supplementation with krill oil significantly decreased levels of P-selectin, sVCAM-1, and NO in serum and the aortic wall thickness compared with those in the high-cholesterol-diet group (*p* < 0.05) (Figure 4). These data indicate that krill oil supplementation can suppress the development of thrombosis and atherosclerosis in hypercholesterolemic rats.

## 3. Discussion

In the present study, we investigated the beneficial effects of krill oil supplementation on hypercholesterolemia to provide a scientific basis for utilizing natural cholesterol-lowering agents. As expected, we found that the high-cholesterol diet increased total triglycerides, total cholesterol, and LDL-cholesterol levels in both serum and liver; however, krill oil supplementation suppressed these effects. In 2004, Bunea et al. demonstrated that administration of krill oil 1–3 g/day reduced the levels of total cholesterol, triglycerides, and LDL in patients with hypercholesterolemia, compared with levels produced by fish oil and a placebo [14]. Moreover, Parolini et al. reported that dietary supplementation of krill oil reduced VLDL and IDL/LDL-cholesterol levels in apoE-deficient mice [15], and Mozaffarian et al. found that a krill-oil-derived ω-3 formulation reduced TG levels in patients with severe hypertriglyceridemia [19]. Accordingly, we observed the factors involved in cholesterol synthesis in the liver to investigate the mechanism of the antihypercholesterolemic effects of krill oil.

We found that the high-cholesterol diet-induced increase in HMG-CoA reductase expression in the liver was decreased in the krill-oil-supplemented groups. Furthermore, krill oil supplementation stimulated AMPK phosphorylation, an inhibitor of HMG CoA-reductase as a kinase phosphorylating the enzyme [20]. Thus, these data indicate that krill oil can inhibit cholesterol synthesis by suppressing HMG-CoA reductase activation via AMPK phosphorylation during a high-cholesterol diet intake. We evaluated the LDL receptor, which is crucial in cellular uptake of apoB-containing lipoproteins, and ACAT2, which plays a role in the esterification of cholesterol storage [21,22]. We found that krill oil supplementation stimulated the LDL receptor and ACAT2 expression in the liver of hypercholesterolemic rats. These results suggest that krill oil supplementation can reduce the levels of total cholesterol and LDL in the blood during hypercholesterolemia by stimulating the uptake of LDL-cholesterol into tissue and via cholesterol esterification. In addition, krill oil supplementation increased the fecal output of cholesterol and bile acid, thereby stimulating cholesterol excretion by blocking reabsorption and promoting bile excretion.

The present study revealed that the krill oil supplementation decreased levels of P-selectin, sVCAM-1, and NO, which activate endothelial cells [23,24]. Furthermore, krill oil supplementation also reduced aortic wall thickness that had been increased by the high-cholesterol diet. Parolini et al. reported that krill-oil-containing diets reduced the plaque area at the aortic sinus, and atherosclerosis development [15]. Therefore, the previous and present studies suggest that krill oil supplementation has a beneficial effect as a natural cholesterol-lowering agent for preventing atherosclerosis, by inhibiting cholesterol synthesis and stimulating cholesterol excretion in hypercholesterolemia.

Hals et al. showed that phospholipids extracted from krill oil were rich in omega-3 fatty acids and demonstrated the effect of krill oil phospholipids on cardiovascular disease risk factors [25]. Therefore, we suggest phospholipids with a high content n−3 LC-PUFA of krill oil may be useful for antihypercholesterolemic effects. Further studies of the pre-clinical and clinical effects of each nutritional component of krill oil are needed to investigate molecular mechanisms.

## 4. Materials and Methods

### 4.1. Extraction of Krill Oil

Krill oil obtained by enzymic hydrolysis (alcalase) was provided by SD Biotechnologies Co., Ltd. (Seoul, Korea). Briefly, frozen or freeze-dried krill was thawed, and salts were removed by washing with tap water. The krill was then pulverized using a pin-type mill. The pulverized krill was mixed with alcalase, a non-specific subtilisin-related serine protease isolated from *Bacillus licheniformis*, and then stirred for 30 to 60 min at room temperature. The enzymatic reaction was performed at 50–60 °C for 3–24 h until liquefaction. After the reaction, the pH of the reactant was adjusted to 3.0–5.0 by adding citric acid and letting it stand for 30 min. The enzyme was inactivated by heating to 95 ± 3 °C. The sludge, including the shells and heads of the krill, was removed by decanter centrifugation (3.0 t/h) at 50–60 °C. Lipids and phospholipids in the filtrate were extracted by centrifugation at 3000–13,000 rpm (1.0–2.0 t/h). The centrifuged extract was purified using pressure filtration. The filtered extract was sterilized and concentrated under reduced pressure using a rotary evaporator at 60–90 °C until the water content dropped below 2%. The sterilized concentrate was filtered through a 50 mesh sieve and stored at room temperature until use. We measured the contents of phospholipids and fatty acid. These consisted of EPA 130.72 mg/g, DHA 77.99 mg/g, and 45% phospholipids (phosphatidylcholine 116.44 ± 6.65 mg, 1-lyso-phosphatidylcholine 2.29 ± 0.60 mg, 2-lyso-phosphatidylcholine 14.83 ± 4.28 mg, phosphatidylethanolamine 1.85 ± 0.36 mg, lyso-phosphatidylethanolamine 0.37 ± 0.04 mg, and N-acyl phosphatidylethanolamine 3.24 ± 0.67 mg).

### 4.2. Animals

The Institutional Animal Care and Use Committee at Kyung Hee University approved the protocol (KHGASP-19-107) for the animal studies. The animals were cared for in accordance with the “Guidelines for Animal Experiments” established by the university.

Six-week-old male Sprague Dawley (SD) rats were purchased from SaeRon Bio (Uiwang, Korea) and housed in cages under automatically controlled temperature (22 ± 2 °C), humidity (about 50%), and lighting (12:12-h light-dark cycle). The rats in the normal diet control group were fed a commercial pelleted chow (AIN 93G rodent purified diet, Orient Bio) and water ad libitum. For the high-cholesterol diet-induced hypercholesterolemia group, the rats were fed a high-cholesterol diet (Table 2; Atherogenic Rodent Diet D12336; Research Diet Inc., New Brunswick, NJ, USA) for 12 weeks. All the rats were randomly divided into five groups of eight rats per group: normal control (NC; AIN 93G diet), control (Cont; high-cholesterol diet), krill oil 100 mg/kg b.w. (KO–L; high-cholesterol diet with Krill oil 100 mg/kg b.w.), and krill oil 200 mg/kg b.w. (KO–H; high-cholesterol diet with Krill oil 200 mg/kg b.w.). Krill oil was administered by oral gavage to mice, using a feeding needle. The amount of food consumption by each group was recorded twice per week for 12 weeks. At the end of the experiment, the mice were sacrificed to collect serum and tissues.

### 4.3. Measurement of Triglyceride, Cholesterols, AST, ALT, HMG-CoA Reductase Activity, ApoB-100, ACAT, P-Selectin, sVCAM-1, and NO

The levels of triglyceride, cholesterols, AST, and ALT in both the serum and liver, and the HMG-CoA reductase activity, ApoB-100, and ACAT in the liver, and P-selectin, sVCAM-1, and NO in the serum were analyzed according to methods described previously [26].

### 4.4. Total RNA Isolation and Real-Time Polymerase Chain Reaction (PCR)

Total RNA was extracted from the livers and analyzed for expression of the genes GAPDH, HMG-CoA reductase, and LDL-receptor by RT-PCR according to methods described previously [26].

### 4.5. Western Blotting

Proteins were extracted from the livers and analyzed for expression phospho-AMP-activated protein kinase (AMPK), HMG-CoA reductase, LDL-receptor, and β-actin according to methods described previously [26].

### 4.6. Measurement of Bile Acid and Cholesterol in Feces

Metabolic cages were designed for appropriate separation of feces and urine through the funnel and separation cone. Initially, metabolic cage food and water baskets were calibrated and filled with water and AIN 93G diet. Rats were individually housed in metabolic cages and monitored daily for access to food and water. After 24 h, feces from each rat were collected separately and subjected to measurement of cholesterol and bile acid contents (Crystal Chem, Elk Grove Village, IL, USA) using commercial kits.

### 4.7. Measurement of Aortic Wall Thickness

The aortas of rats were fixed in 10% paraformaldehyde and embedded in paraffin. Paraffin blocks were cut into 5 µm slices; the sections were then stained with hematoxylin and eosin, and observed using a light microscope.

### 4.8. Statistical Analysis

All data are presented as mean ± standard deviation (SD). The data were statistically evaluated using Duncan’s multiple range tests after one-way ANOVA using SPSS statistical procedures (SPSS PASW Statistic 23.0, SPSS Inc., Chicago, IL, USA). Statistically significant differences were considered at the *p* < 0.05 level.

## 5. Conclusions

In the present study, we investigated the antihypercholesterolemic effects of krill oil supplementation by observing its effect on cholesterol synthesis and excretion in high-cholesterol diet-induced hypercholesterolemic rats. We demonstrated that krill oil supplementation can reduce the levels of LDL-cholesterol in the blood during hypercholesterolemia by stimulating both the uptake of LDL-cholesterol into tissue and cholesterol excretion, and inhibiting cholesterol synthesis. In addition, supplementation with krill oil decreased levels of P-selectin, sVCAM-1, and NO in serum and aortic wall thickness. These results indicate that krill oil has a protective effect against hypercholesterolemia and atherosclerosis development at a dose of 100–200 mg/kg b.w. in the rat. This study provides scientific evidence for, and describes the mechanisms underlying, the antihypercholesterolemic effects of krill oil.

## Figures and Tables

**Table 1:** Change in body and tissue weights, and serum ALT and AST, of hypercholesterolemic rats fed a diet supplemented with krill oil.

	NC	Control	KO–L	KO–H
Weight gain (g)	267.25 ± 19.15 ^b^	355.38 ± 41.14 ^a^	325.59 ± 11.84 ^a^	319.43 ± 31.68 ^a^
Food consumption (g/day)	21.36 ± 1.09 ^a^	20.42 ± 0.88 ^b^	19.48 ± 0.83 ^b^	19.62 ± 1.04 ^b^
FER	16.17 ± 1.99 ^b^	19.31 ± 0.94 ^a^	20.28 ± 0.74 ^a^	19.90 ± 1.97 ^a^
Organ weight (g)/body weight (g) × 100				
Kidney	0.60 ± 0.02 ^ab^	0.67 ± 0.15 ^a^	0.57 ± 0.03 ^b^	0.59 ± 0.03 ^ab^
Spleen	0.16 ± 0.02 ^c^	0.28 ± 0.03 ^a^	0.23 ± 0.04 ^b^	0.21 ± 0.03 ^b^
Liver	3.78 ± 0.32 ^c^	6.15 ± 0.34 ^a^	5.84 ± 0.23 ^ab^	5.68 ± 0.28 ^b^
Serum ALT (mU/mL)	68.27 ± 11.38 ^c^	101.73 ±19.37 ^a^	79.83 ± 12.73 ^b^	81.09 ± 12.84 ^b^
Serum AST (mU/mL)	403.08 ± 24.11 ^c^	894.59 ± 56.07 ^a^	793.54 ± 34.98 ^b^	764.43 ± 108.60 ^b^

NC, normal AIN93G diet control group; Control, high-cholesterol diet control group; KO–L, high-cholesterol diet with krill oil 100 mg/kg b.w.; KO–H, high-cholesterol diet with krill oil 200 mg/kg b.w. Values are presented as means ± SD. Different letters indicate a significant difference at *p* < 0.05, as determined by Duncan’s multiple range test.

**Table 2:** Formulation of high-cholesterol diet.

Ingredient	gm	kcal
Soy Protein	130	520
Casein, Lactic, 30 Mesh	75	300
Methionine, DL	2	8
Starch, Corn	275	1100
Maltodextrin	150	600
Sucrose	30	120
Cellulose, BW200	90	0
Soybean Oil, USP	50	450
Cocoa Butter	75	675
Coconut Oil, 76	35	315
Mineral Mix S10001	35	0
Calcium Carbonate	5.5	0
Sodium Chloride	8	0
Potassium Citrate	10	0
Mineral Mix V10001	10	40
Choline Bitartrate	2	0
Cholesterol, NF	12.5	0
Sodium Cholic Acid	5	0
FD and C Red Dye #40	0.1	0
Total	1000.1	4128

## Data Availability

Not applicable.

## Supplementary Materials

The following are available online at https://www.mdpi.com/article/10.3390/md20100609/s1, Figure S1. Quantification of the western blot bands relative expression in Figure 2D.
