# Supplementary figures and images for: Krill Oil Inhibits Cholesterol Synthesis and Stimulated Cholesterol Excretion in Hypercholesterolemic Rats
Source: Mar Drugs. 2022 Sep 27;20(10):609. doi: 10.3390/md20100609 (PMC9605538; doi:10.3390/md20100609)

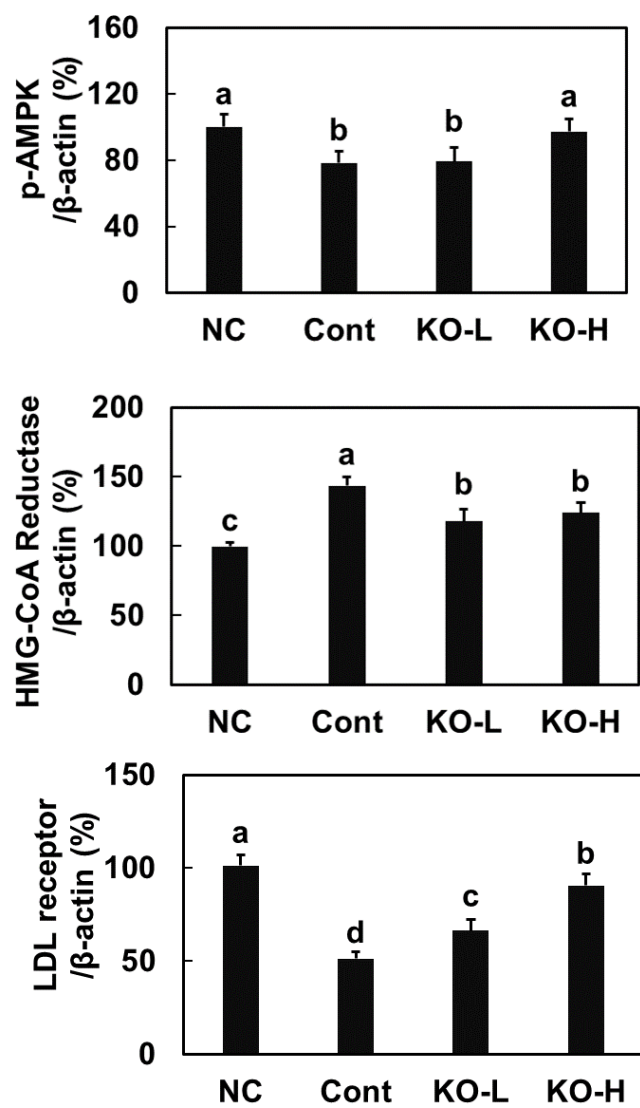

Figure S1. Quantification of the western blot bands relative expression in Figure 2D.

Supplement: Supplementary file 1 [file marinedrugs-20-00609-s001.zip › marinedrugs-1903893-supplementary.pdf]
